# Supplementary material for: Mice lacking the mitochondrial exonuclease MGME1 accumulate mtDNA deletions without developing progeria
Source: Nat Commun. 2018 Mar 23;9:1202. doi: 10.1038/s41467-018-03552-x (PMC5865154; doi:10.1038/s41467-018-03552-x)
Supplement: Supplementary file 1 — Supplementary Information [file 41467_2018_3552_MOESM1_ESM.pdf]

SUPPLEMENTARY INFORMATION

Mice lacking the mitochondrial exonuclease *MGME1* accumulate mtDNA deletions without developing progeria

"Matic et al."

SUPPLEMENTARY METHODS

Mapping of DNA ends by strand-specific sequencing, LM-PCR and 3′ polyadenylation-mediated PCR amplification

To capture the 5′ ends of the 7S DNA by LM-PCR a DNA adaptor (5′-GCTGATGGCGATGAATGAACACTGCGTTTGCTGGCTTTGATGAAA-3′) was ligated to purified mtDNA using T4 RNA Ligase (New England Biolabs), purified using a Zymo DNA Clean and Concentrator kit, and fragments amplified by PCR using the following primers: 5′-CGCGGATCCGAACACTGCGTTTGCTGGCTTTGATG-3′ and 5′-ATCTGGTTCTTACTTCAGGGCCATC-3′. LM-PCR to identify the 3′ ends of the 7S DNA used T4 RNA ligase to attach a linker oligo (5′-Phos-ACCTATAGTGAGTCGTATTAATTCTGTGCTCGC-C3 spacer-3′) to purified mtDNA, which was then purified using a Zymo DNA Clean and Concentrator kit, and fragments amplified by PCR using the following primers: 5′-CGCGGATCCGAATTAATACGACTCACTATAGG-3′ and 5′-GTTTAGCTACCCCCAAGTTTAATGG-3′. 3′ ends of the 7S DNA were captured by 3′ polyadenylation-mediated PCR amplification using terminal transferase (New England Biolabs) to polyadenylate purified mtDNA and an oligo(dT) primer was used to make the DNA double stranded (5′-GCGAGCACAGAATTAATACGACTCACTATAGGTTTTTTTTTTTTVN-3′). After purification using a Zymo DNA Clean and Concentrator kit, fragments were amplified by PCR using the following primers: 5′-CGCGGATCCGAATTAATACGACTCACTATAGG-3′ and 5′-GTTTAGCTACCCCCAAGTTTAATGG-3′. Products were resolved on a 2% agarose gel and cloned using a Thermo Scientific CloneJET PCR Cloning kit.

MtDNA mutation load analysis. mtDNA mutation load analysis was performed as previously described[58]. Briefly, a post-PCR cloning and sequencing method was used to assay mutation loads. Mutations were analyzed by cloning (Zero Blunt TOPO PCR Cloning Kit, Invitrogen) of an mtDNA fragment (containing WANCY cluster of tRNAs) amplified with a high-fidelity polymerase and individual clones were sequenced with M13 primers (PlateSeq service, Eurofins).

Histology

Tissues were fixed in 10% neutral Formalin (spleen) or in Bouin's fixative solution (testes), sequentially dehydrated and paraffin-embedded. Sections (5 µm) were cut, mounted on glass slides, deparaffinized with xylene and stained with hematoxylin and eosin (HE) for light microscope observations.

Blood count

Mice were sacrificed and blood was collected into EDTA tube by heart puncture. Blood count was performed using whole blood samples by the analyzer from Laboklin (Labor für Klinische Diagnostik).

SUPPLEMENTARY FIGURES

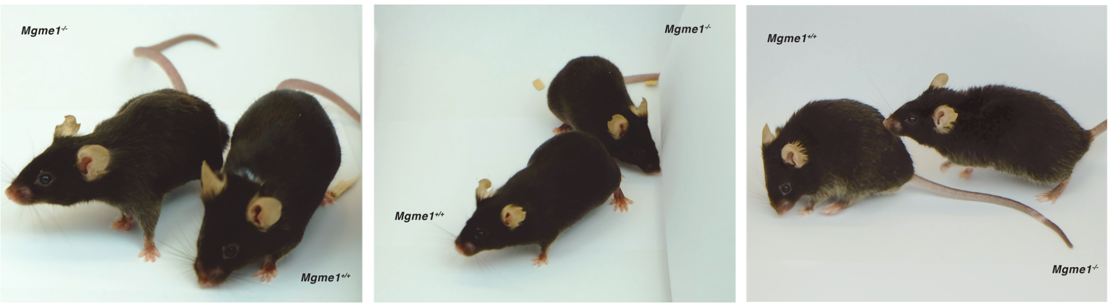

Supplementary Figure 1. Appearance of *Mgme1* knockout mice at 70 weeks of age. Left and middle panel shows female animals and the panel on the right male animals at 70 weeks of age. Wild-type (*Mgme1*$^{+/+}$); *Mgme1* knockout mice (*Mgme1*$^{-/-}$).

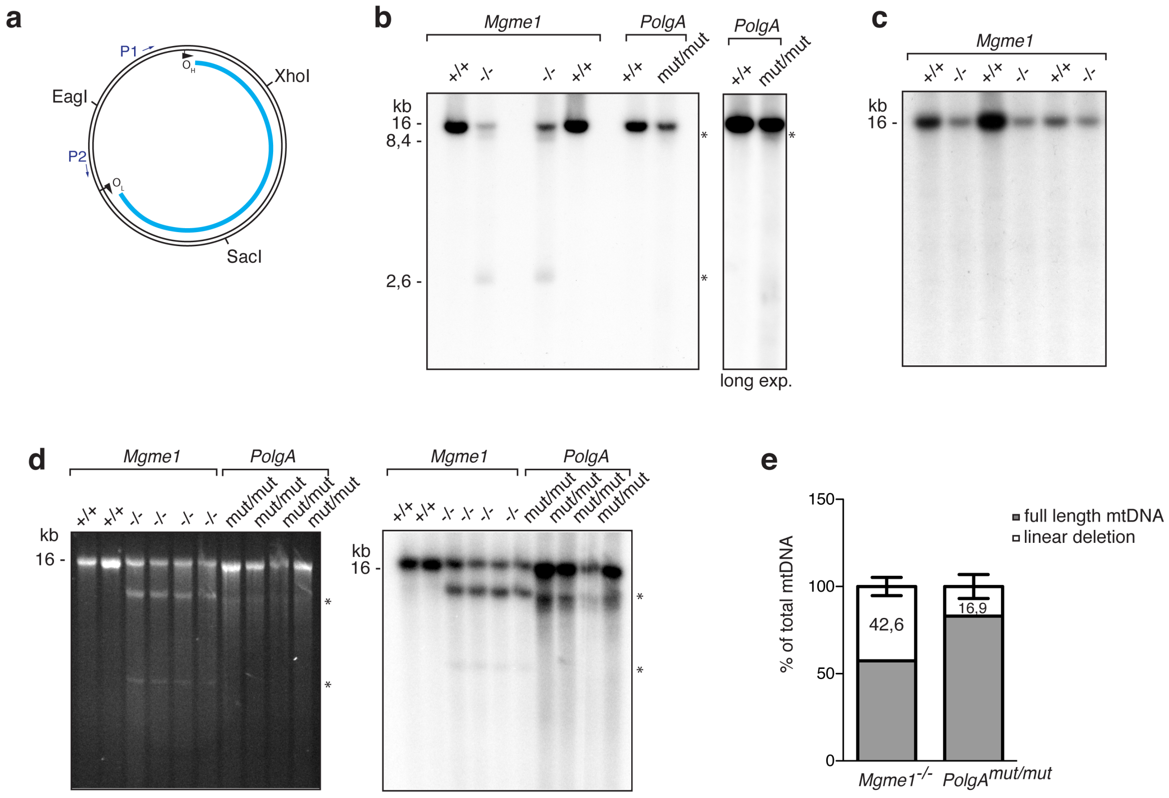

Supplementary Figure 2. Mapping of mtDNA linear deletion. (**a**) Schematic representation of mtDNA molecule with indicated positions of restriction enzyme recognition sites. Liner deletion is represented in blue. P1 and P2, primers used for Long-extension PCR (**b**) Southern blot analysis of *XhoI*-digested heart DNA from wild-type (*Mgme1*$^{+/+}$) and *Mgme1* knockout mice (*Mgme1*$^{-/-}$) as well as control (*PolgA*$^{+/+}$) and mtDNA-mutator mice (*PolgA*$^{mut/mut}$). Plasmid pAM1 was used as a probe. * linear deletion **(c)** Southern blot analysis of *EagI*-digested heart DNA from wild-type (+/+) and *Mgme1* knockout mice (-/-) at 8 weeks of age. Probe from 12S ribosomal RNA was used for the labeling. (**d**) Southern blot analysis of *SacI*-digested liver mtDNA from wild-type (*Mgme1*$^{+/+}$) and *Mgme1* knockout mice (*Mgme1*$^{-/-}$) as well as mtDNA-mutator mice (*PolgA*$^{mut/mut}$). Left panel, EtBr stained gel; right panel, Southern blot membrane was labelled using cytb as a probe. * linear deletion (e) Southern blot quantification: % of linear deletion to total mtDNA. Student's *t*-test. Error bars represent SEM.

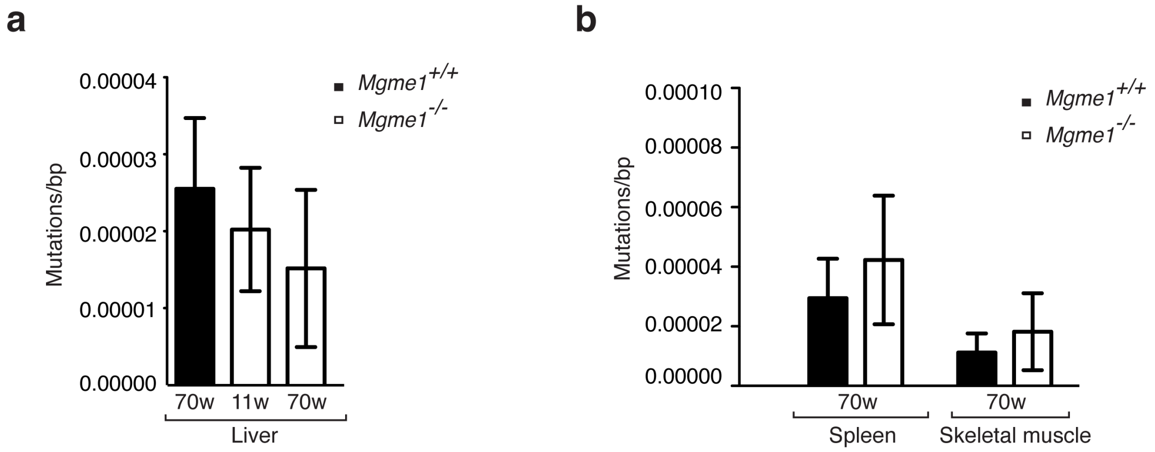

Supplementary Figure 3. Quantitative assessment of mtDNA point mutation in various tissues of *Mgme1*$^{-/-}$ mice. (**a**) Post-PCR cloning and sequencing was used to quantify mtDNA mutation load in liver of 70 weeks old controls and 11 weeks and 70 weeks old *Mgme1*$^{-/-}$mice (**b**) as (**a**) except that spleen and skeletal muscle tissue from 70 weeks old controls and *Mgme1*$^{-/-}$mice was used for the analysis. For each point and tissue three animals were analyzed. Holm-Sidak's multiple comparisons test. Error bars represent SEM.

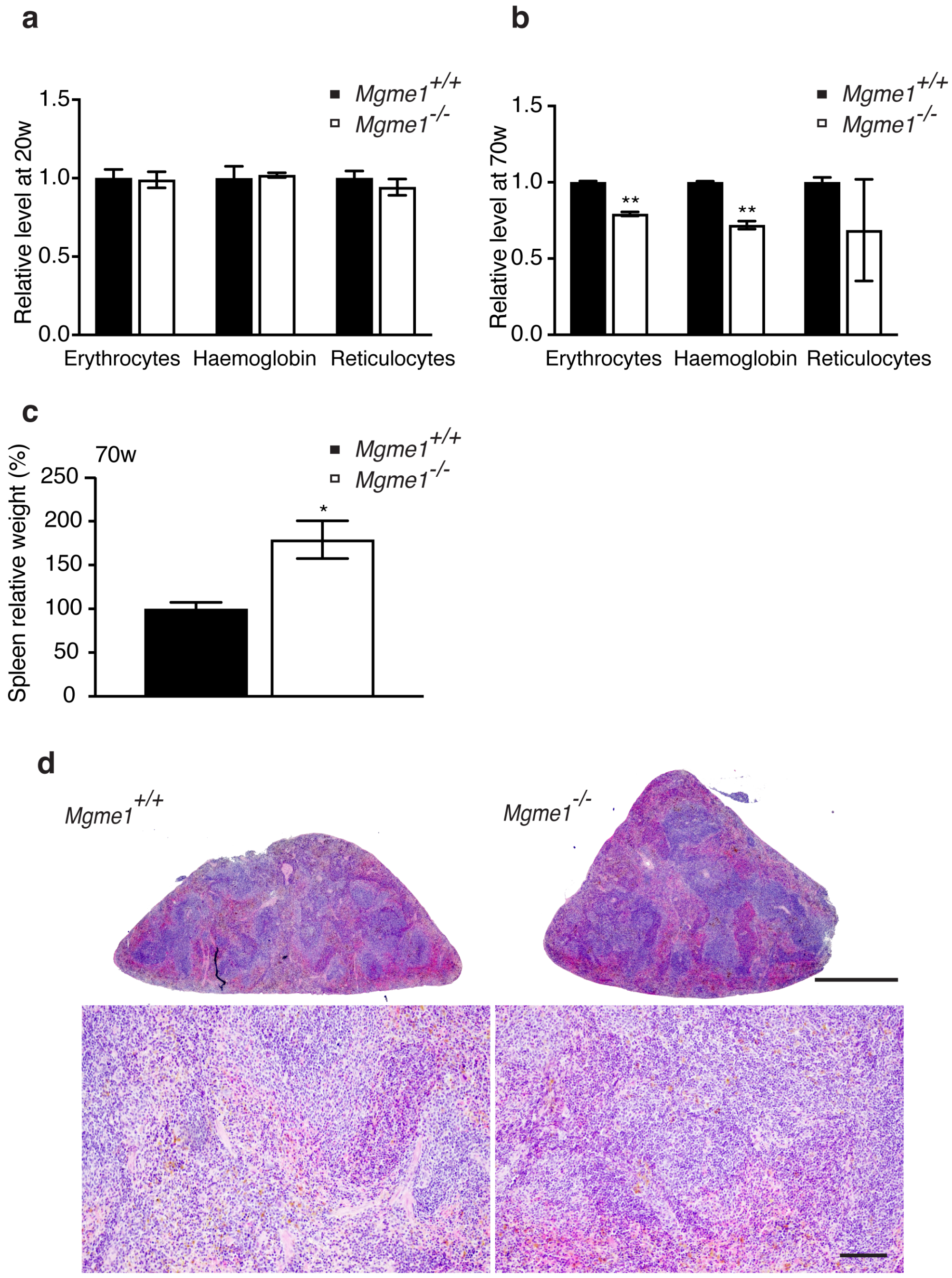

Supplementary Figure 4. Blood count analysis and spleen histology of *Mgme1*$^{-/-}$ mice. Blood count of 20 weeks (**a**) and 70 weeks old (**b**) *Mgme1*$^{-/-}$ and control mice. (**c**) Relative spleen weight of 70 weeks old *Mgme1*$^{-/-}$ and control mice. Data are represented as mean ± SEM. n=3 biological repeats. Student's t test. $^{*}P < 0.05$; $^{**}P < 0.01$. (**d**) HE staining of spleen of 70 weeks old *Mgme1*$^{-/-}$ and control mice. Upper panel scale bar: 1mm. Lower panel scale bar: 100μm.

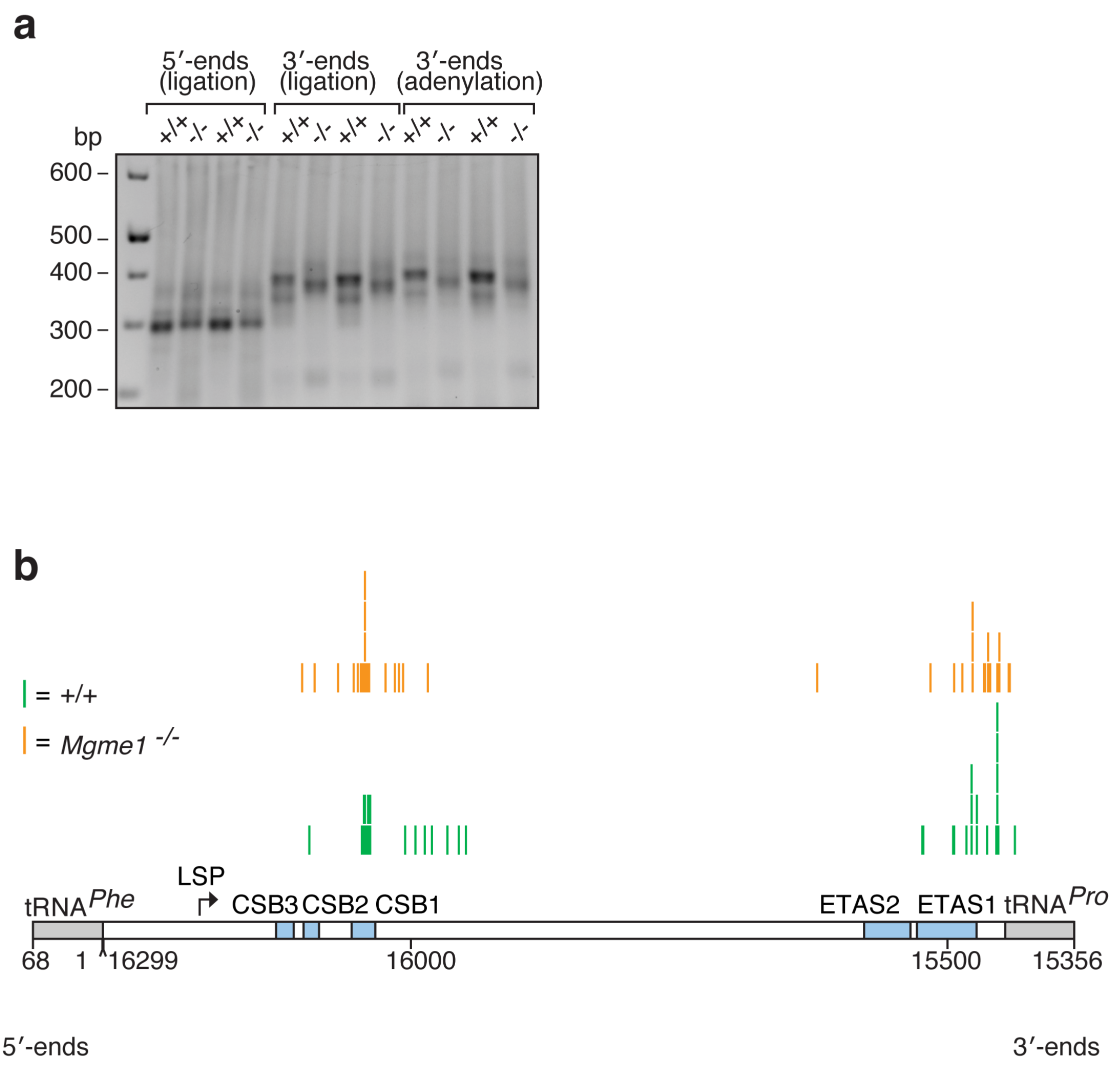

Supplementary Figure 5. Mapping of 7S DNA ends in Mgme1 knockout mice. (**a**) Agarose gel electrophoresis of LM-PCR and 3′ polyadenylation-mediated PCR amplification products from wild type (+/+) and Mgme1 knockout (-/-) heart samples. (**b**) Schematic representation of 3′ and 5′ ends obtained by sequencing of cloned PCR products. Each vertical line represents single clone that was sequenced. Conserved sequence elements present at 3′ (CSB1-3) and 5′ NCR termini (ETAS1 and ETAS2) are shown.

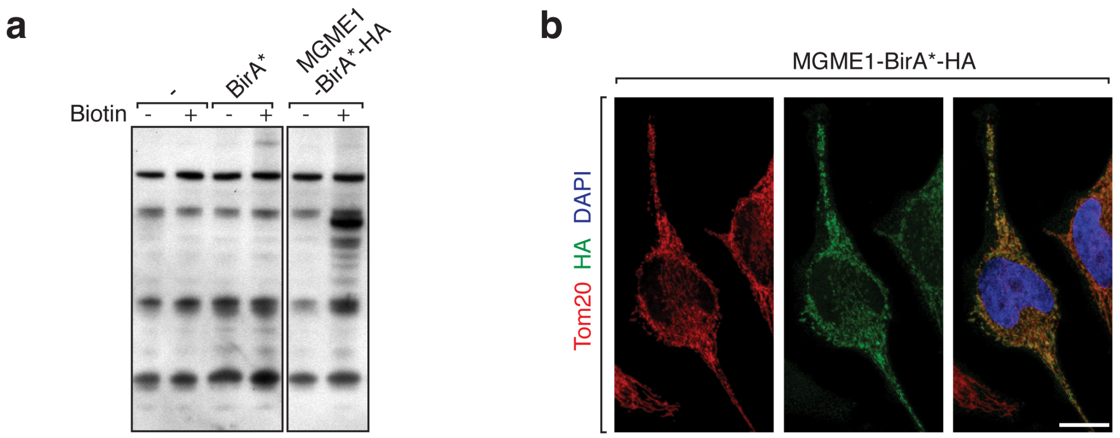

Supplementary Figure 6. BirA* does not interfere with MGME1 localization. (**a**) Biotinylation efficiency of MGME1-BirA*. BioID fusion protein was expressed in HeLa cells by transient transfection. Cells were either treated to a final concentration of 50μM biotin or untreated. Total cell protein extracts were separated by SDS-PAGE and visualized by streptavidin HRP. (**b**) Immunohistochemical analysis of subcellular localization of MGME1-BirA*-HA fusion protein. Fusion protein is detected with fluorescently labeled anti-HA antibody. Mitochondria were labeled with TOM20 as a marker and nuclei were stained with DAPI, respectively. Scale bars represent 10 μm.

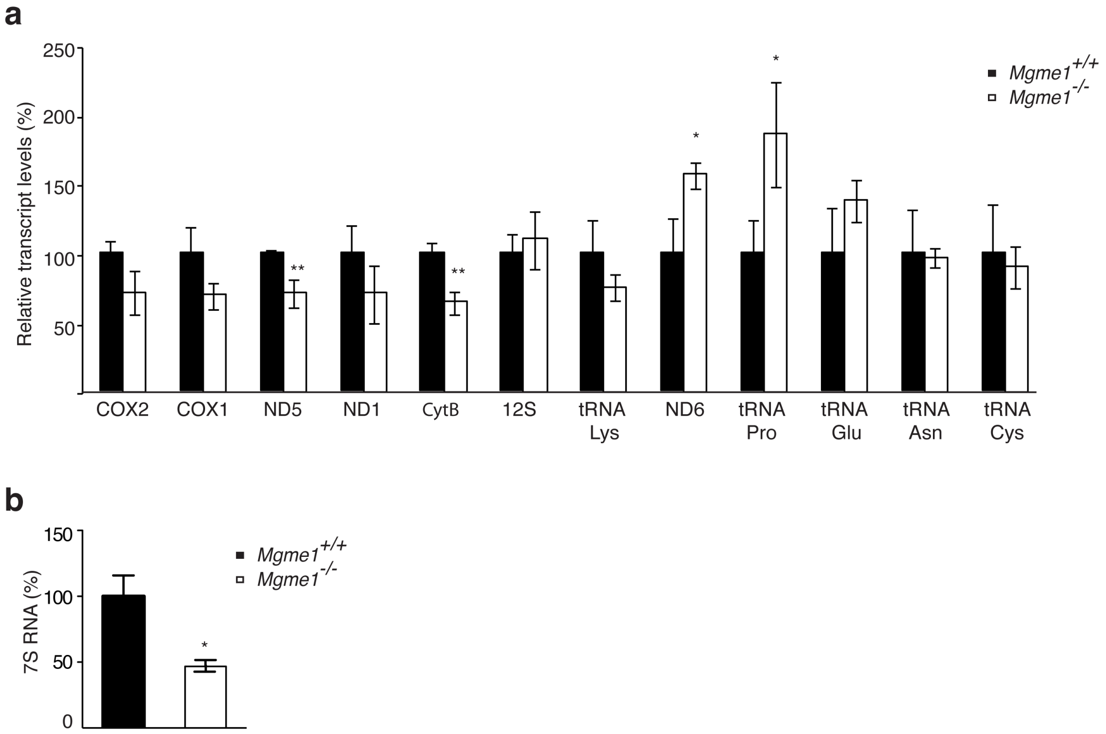

Supplementary Figure 7. Quantification of steady-state levels of mitochondrial transcripts in absence of MGME1. (**a**) Steady-state levels of mRNAs and tRNAs in wild type (+/+) and Mgme1 knockout (-/-) heart samples. n=3. Student's t test. Error bars represent the SEM. * P< 0,05; ** P< 0,001. (**b**) Steady-state levels of 7S RNA in wild type (+/+) and Mgme1 knockout (-/-) heart samples. n=3. Student's t test. Error bars represent SEM. * P< 0,05.

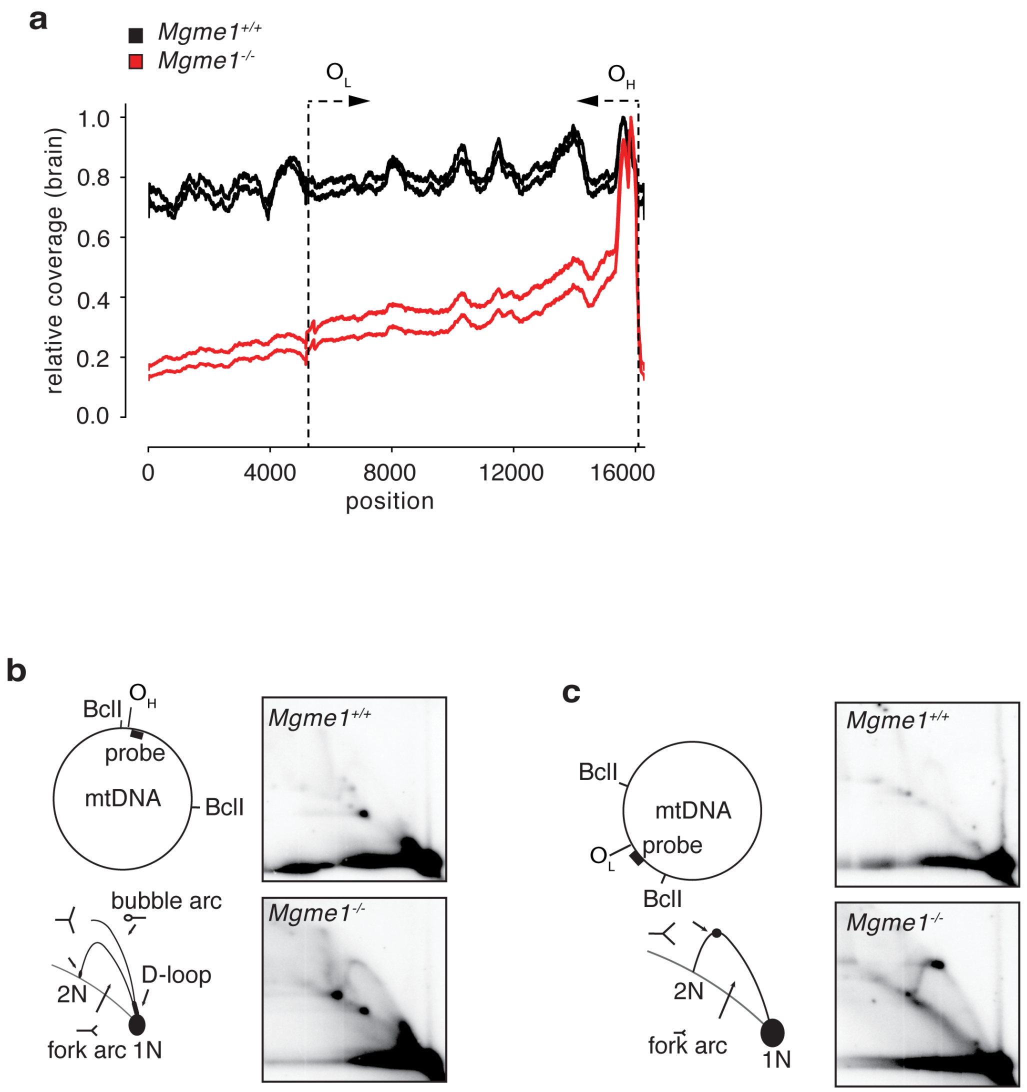

Supplementary Figure 8. Replication stalling in brain of Mgme1 knockout mice visualized by next generation sequencing and 2DNAGE. (**a**) Sequence coverage of the mtDNA samples from brain of *Mgme1* knockout mice (-/-) and controls (+/+). Mitochondrial genome position (x-axis) versus sequence coverage divided by maximum coverage for each sample. Number of lines corresponds to the sample number. The approximate locations of the origins of light-strand (O_L) and heavy-strand (O_H) replication are indicated by dotted lines with arrows. (**b**) and (**c**) mtDNA replication pattern in the brain Mgme1$^{+/+}$ and Mgme1$^{-/-}$ mice analyzed by two-dimensional neutral-agarose gel electrophoresis followed by Southern blot. Restriction enzymes and probes used are indicated to the left. The black bars indicate probe used, and O_H marks the origin of H-strand replication and O_L denotes the origin of L-strand replication. 1N indicates the migration of a fragment of non-replicating mtDNA, and 2N indicates the migration of fully-replicated mtDNA

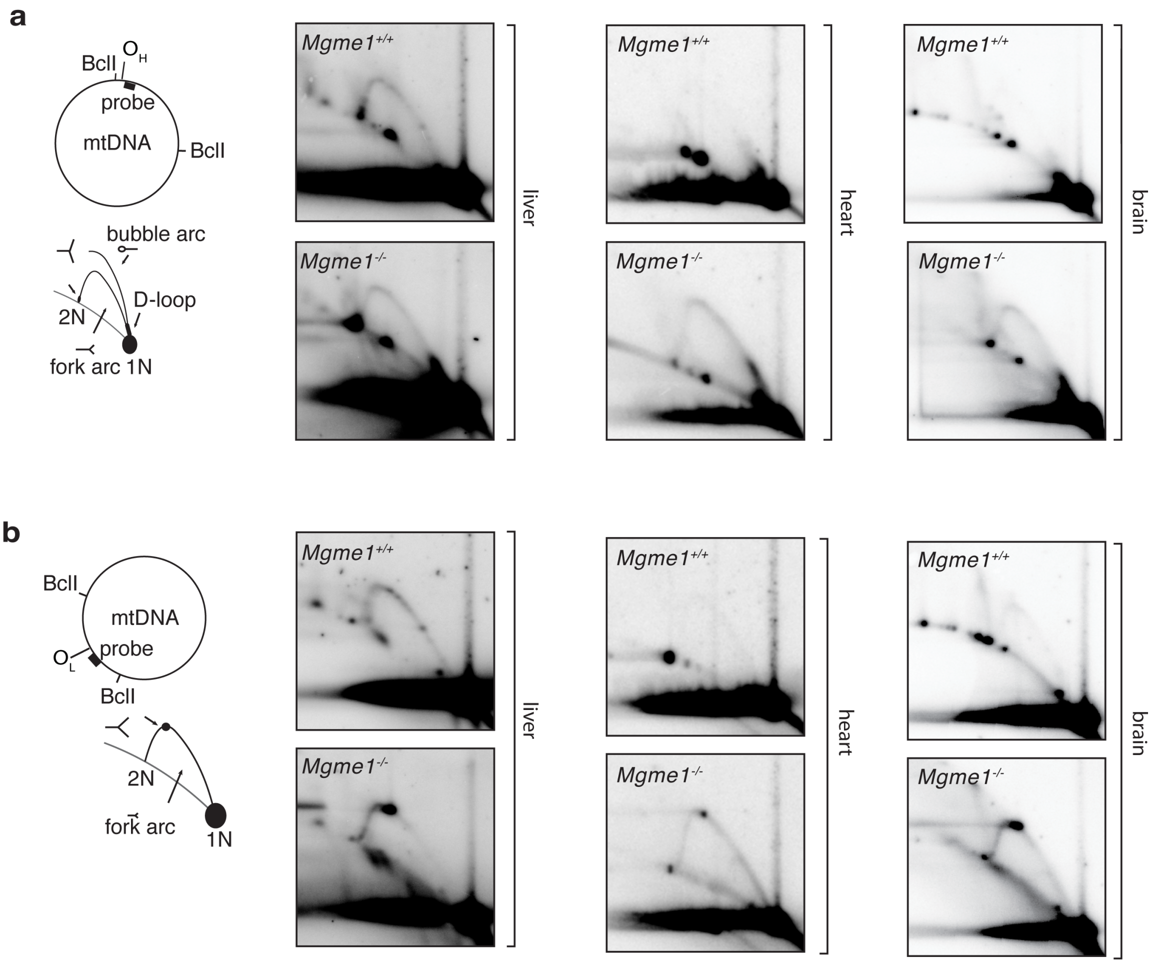

Supplementary Figure 9. | Replication stalling in liver, heart and brain of Mgme1 knockout mice visualized by 2DNAGE. mtDNA replication pattern in the liver, heart and brain $Mgme1^{+/+}$ and $Mgme1^{-/-}$ mice analyzed by two-dimensional neutral-agarose gel electrophoresis followed by Southern blot. Restriction enzymes and probes used are indicated to the left. The black bars indicate probe used, and O_H marks the origin of H-strand replication and O_L denotes the origin of L-strand replication. 1N indicates the migration of a fragment of non-replicating mtDNA, and 2N indicates the migration of fully-replicated mtDNA.

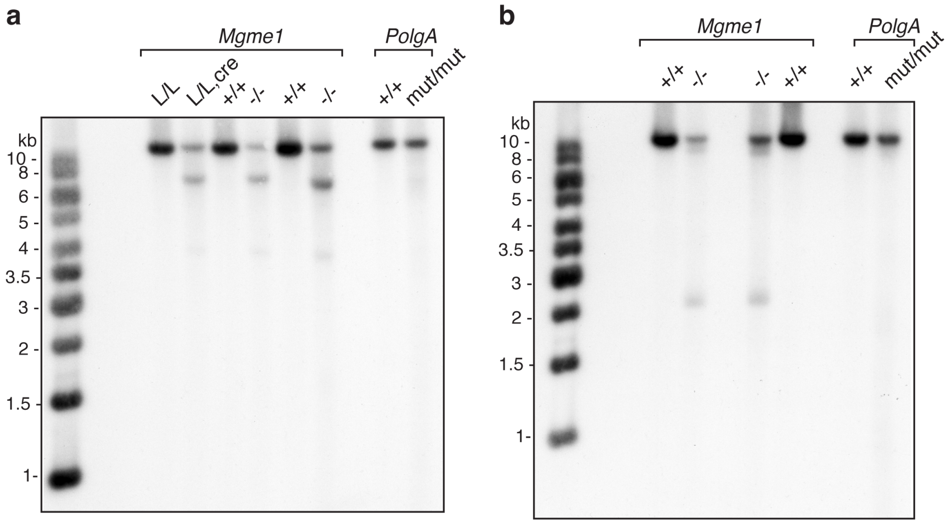

Supplementary Figure 10. | Full size Southern blot images including marker lanes. Related to Fig. 2b (**a**) and Supplementary Fig. 1b (**b**).
