# Supplementary material for: Mice lacking the mitochondrial exonuclease MGME1 accumulate mtDNA deletions without developing progeria
Source: Nat Commun. 2018 Mar 23;9:1202. doi: 10.1038/s41467-018-03552-x (PMC5865154; doi:10.1038/s41467-018-03552-x)
Supplement: Supplementary file 2 — Description of Additional Supplementary Files [file 41467_2018_3552_MOESM2_ESM.pdf]

Description of Additional Supplementary Files

File Name: Supplementary Data 1
Description: List of proteins identified in pull-down by MGME1-BirA* (See enclosed Excel-File).

File Name: Supplementary Data 2
Description: List of probes and antibodies.
